# Differential methylation of interferon-related genes is associated with autoantibody production in systemic lupus erythematosus

**DOI:** 10.1186/ar4625

**Published:** 2014-09-18

**Authors:** Sharon A Chung, Joanne Nititham, Emon Elboudwarej, Hong L Quach, Kimberly E Taylor, Lisa F Barcellos, Lindsey A Criswell

**Affiliations:** 1 Rosalind Russell/Ephraim P. Engleman Rheumatology Research Center, University of California, San Francisco, CA, USA; 2 School of Public Health, University of California, Berkeley, CA, USA

## Background

DNA methylation, an epigenetic modification, influences gene expression and has been implicated in the pathogenesis of systemic lupus erythematosus (SLE). Two recent studies suggest that interferon-regulated genes are hypomethylated in SLE patients compared with unaffected controls. However, these studies have not examined whether DNA methylation is associated with specific disease manifestations. The goal for the current study was to determine whether differential DNA methylation is associated with autoantibody production in SLE, with a focus on the anti-dsDNA autoantibody.

## Methods

The methylation status of 467,314 CpG sites across the genome was characterized for 326 women with SLE. Associations between anti-dsDNA autoantibody production and methylation status was assessed using a discovery and replication study design. Multivariable regression was used to adjust for confounders including estimated leukocyte cell proportions and population substructure. In secondary analyses, we assessed differential methylation associations with anti-SSA, anti-Sm, and anti-RNP autoantibody production.

## Results

Significant associations between anti-dsDNA autoantibody production and methylation status were replicated for 16 CpG sites (*P*_discovery _< 1.07 × 10^-7 ^and *P*_replication _< 0.0029) in 11 genes. The adjusted mean difference in methylation between the two autoantibody subgroups ranged from 1 to 19%, and the adjusted odds ratio for anti-dsDNA autoantibody production comparing the lowest with the highest tertile of methylation ranged from 6.8 to 18.2. Differential methylation for these sites was also associated with anti-SSA, anti-Sm, and anti-RNP autoantibody production. All associated sites were less methylated in autoantibody-positive compared with autoantibody-negative cases. Seven of the 11 associated genes either induce interferon or regulate the interferon signaling pathway. Among the differentially methylated genes associated with the production of at least one autoantibody, cytokine and interferon signaling pathways were the most represented.

## Conclusions

Hypomethylation of interferon-related and other genes is associated with autoantibody production among SLE cases. Differential methylation of these 11 genes in autoantibody-positive SLE cases may explain their recently reported associations with SLE risk, and the extent of hypomethylation may influence disease manifestations.

